# Psychological factors contributing to vocal cord dysfunction in pediatric population pre-pandemic and during pandemic

**DOI:** 10.3389/fped.2026.1717883

**Published:** 2026-02-18

**Authors:** Aledie Navas Nazario, Sreekara Singam, Zhuo Li, Carolyn Rapp, Floyd Livingston

**Affiliations:** 1 Nemours Children’s Health, Orlando, FL, United States; 2 Baylor University Medical Center, Dallas, TX, United States; 3 Mayo Clinic, Jacksonville, FL, United States; 4 University of North Carolina, Chapel Hill, NC, United States

**Keywords:** anxiety and depression, COVID-19 pandemic, health care utilization, induciblelaryngeal obstruction, pediatric asthma misdiagnosis, psychological comorbidities, retrospective chart review, vocal cord dysfunction (VCD)

## Abstract

**Background:**

Vocal cord dysfunction (VCD) is an underrecognized differential diagnosis for asthma and is often influenced by psychological factors. The COVID-19 pandemic introduced new stressors and disrupted access to pediatric care, potentially affecting VCD incidence and recognition.

**Objectives:**

This study aimed to determine whether the incidence of pediatric VCD at Nemours Children's Hospital in Orlando changed during the COVID-19 pandemic and to identify psychological diagnoses most associated with VCD.

**Methods:**

A retrospective chart review was conducted for patients diagnosed with VCD between January 2017 and July 2022, including 2.5 years before and 2.5 years during the pandemic. Demographics, diagnostic methods, triggers, comorbidities, and psychological conditions were extracted from the electronic medical record.

**Results:**

Among 74,022 patients (45,199 pre-pandemic; 28,823 pandemic), VCD incidence significantly decreased during the pandemic (0.7% vs. 0.3%, *p* < 0.001). Psychological diagnoses also declined modestly: anxiety (2.6% vs. 2.0%), depression (0.3% vs. 0.2%), and ADHD (4.4% vs. 3.5%). Compared with non-VCD patients, those with VCD were older (median 14 vs. 9 years), predominantly female (71% vs. 47%), and more often White/non-Hispanic. They had higher rates of asthma (41% vs. 16%), allergic rhinitis (20% vs. 11%), gastroesophageal reflux (31% vs. 4%), and psychological conditions, including anxiety (8.5% vs. 2.3%), depression (1.5% vs. 0.2%), and panic attacks (0.8% vs. 0.1%).

**Conclusions:**

In contrast to prior reports, VCD incidence declined during the pandemic, likely reflecting reduced healthcare access or underdiagnosis. Persistent associations with psychological conditions highlight the biopsychosocial nature of VCD and the importance of multidisciplinary evaluation in pediatric populations.

## 1 Introduction

Vocal cord dysfunction (VCD) is an underrecognized differential diagnosis for asthma. Patients with VCD frequently present with episodes of acute dyspnea, cough, stridor, wheeze, and/or chest tightness, often leading to misdiagnosis and treatment for asthma that is refractory to standard interventions. This diagnostic delay can result in repeated medical visits, unnecessary health care utilization, and increased costs over extended periods (1–3).

Psychological conditions are considered important contributors to VCD, as described in case reports involving patients with a history of sexual abuse, elite athletes, or individuals experiencing significant social stressors (4–8). The COVID-19 pandemic had a profound impact on mental health, particularly among pediatric populations (9). Recent narrative reviews have further emphasized that youth are among the groups at highest risk for pandemic-related mental health sequelae, including depression, anxiety, suicidality, and impaired cognitive functioning (10–12). These findings underscore the importance of evaluating psychological comorbidities in conditions such as VCD.

Case studies have further suggested a potential causal link between COVID-19 and VCD, even in the absence of psychological comorbidities (10). In contrast, a recent study by Edge et al. reported an increased incidence of VCD during the COVID-19 pandemic, although psychological diagnoses were not assessed (12).

At the same time, the COVID-19 pandemic introduced substantial heterogeneity in children's psychological and environmental exposures, with some stressors intensifying while others, such as school-related pressures and athletic participation, were reduced. Disruptions in routine pediatric care during this period further limited access to preventive and outpatient visits, particularly among U.S. children ≤12 years of age, which likely delayed the recognition and diagnosis of conditions such as VCD and associated psychological comorbidities. Together, these overlapping changes in psychosocial stressors and healthcare access complicate interpretation of observed trends in VCD during the pandemic and underscore the need for a clearer understanding of its epidemiology during this unique period.

We therefore conducted a retrospective chart review to determine whether the incidence of VCD increased during the pandemic and to identify the most common psychological diagnoses associated with VCD in a pediatric population.

### 1.1 Objectives

The primary aim of this study was to determine whether the incidence of VCD in the pediatric population at Nemours Children's Hospital (NCH) increased during the COVID-19 pandemic. The secondary aim was to identify the most common psychological diagnoses associated with VCD at NCH.

## 2 Design and methods

We conducted a retrospective chart review of patients diagnosed with VCD at NCH between January 2017 and July 2022 after IRB approval (1938006-3) was obtained. The study period included 2.5 years prior to the pandemic and 2.5 years during the pandemic. Data were extracted from the EPIC electronic health record system and stored in a password-protected Excel file on the Nemours Pediatric Pulmonology Drive.

Collected variables included demographic information (age, sex, ethnicity), date of VCD diagnosis [pre-pandemic [07/01/2017–01/20/2020] or pandemic [01/21/2020–07/31/2022]] diagnostic methods used (spirometry, laryngoscopy, clinical diagnosis), identified triggers, number of VCD episodes (recorded as pre-pandemic or post pandemic), presence of psychological diagnoses (anxiety, depression, attention deficit hyperactivity disorder, obsessive compulsive disorder, tic disorder) and date of diagnosis (pre- or post-pandemic), history of sexual or substance abuse, exposure to vaping and electronic cigarettes, and other associated medical conditions including allergic rhinitis, asthma, gastroesophageal reflux, laryngopharyngeal reflux, and post-nasal drip.

Vocal cord disorder was identified based on clinician-documented diagnosis in the electronic medical record, supported by characteristic clinical features and, when available, laryngoscopic findings. Psychological comorbidities, including anxiety, depression, and panic disorder, were defined by documented clinical diagnoses recorded in the medical record.

Chi-square analyses were used to compare the incidence rate of VCD between the two time periods. The association between psychological diagnosis and VCD was also tested using chi-square analysis. All tests were two-sided with *p*-value < 0.05 considered as statistically significant. The analysis was done using R4.1.2.

## 3 Results

### 3.1 Demographic characteristics

The study population included 45,199 patients pre-pandemic and 28,823 patients during the pandemic. The median age was 9 years in both groups, with the mean age unchanged at 9.8 years. Sex distribution remained similar, with males slightly more represented overall. Ethnic distribution shifted modestly, with Hispanic/Latino patients comprising a higher proportion during the pandemic (20.1% vs. 18.4%). Racial distribution showed small but statistically significant differences, although White/Caucasian patients remained the majority (Table 1).

**Table 1:** Patient demographic data.

Demographics	Before pandemic	During pandemic	Total	*P* value
		(*N* = 28,823)	(*N* = 74,022)	
AGE				<0.001
Median (Range)	9.0 (5.0, 21.0)	9.0 (5.0, 21.0)	9.0 (5.0, 21.0)	
Mean (SD)	9.8 (3.9)	9.8 (4.0)	9.8 (3.9)	
SEX				0.031
F	20,933 (46.3%)	13,582 (47.1%)	34,515 (46.6%)	
M	24,266 (53.7%)	15,241 (52.9%)	39,507 (53.4%)	
ETHNICITY				<0.001
N-Miss	1,724	1,421	3,145	
ANOTHER HISPANIC, LATINO, OR SPANISH ORIGIN	7,978 (18.4%)	5,518 (20.1%)	13,496 (19.0%)	
NON-HISPANIC OR LATINO	35,497 (81.6%)	21,884 (79.9%)	57,381 (81.0%)	
RACE				0.002
WHITE OR CAUCASIAN	26,352 (58.3%)	16,632 (57.7%)	42,984 (58.1%)	
BLACK OR AFRICAN AMERICAN	8,310 (18.4%)	5,591 (19.4%)	13,901 (18.8%)	
OTHER	10,537 (23.3%)	6,600 (22.9%)	17,137 (23.2%)	

F, female; M, male; SD, standard deviation.

### 3.2 Incidence of VCD and psychological diagnoses

The incidence of VCD decreased significantly from 0.7% pre-pandemic to 0.3% during the pandemic (*p* < 0.001). Psychological diagnoses also showed modest declines: depression (0.3% to 0.2%), anxiety (2.6% to 2.0%), and ADHD (4.4% to 3.5%). Bipolar disorder, mood disorder, and adjustment disorder remained rare but demonstrated statistically significant differences due to the large sample size (Table 2).

**Table 2:** Incidence rate of VCD and psychological diagnosis before and during pandemic.

Diagnoses	Before pandemic	During pandemic	Total	*P* value
	(*N* = 45,199)	(*N* = 28,823)	(*N* = 74,022)	
VCD				<0.001
0	44,897 (99.3%)	28,726 (99.7%)	73,623 (99.5%)	
1	302 (0.7%)	97 (0.3%)	399 (0.5%)	
DEPRESSION				0.002
0	45,081 (99.7%)	28,779 (99.8%)	73,860 (99.8%)	
1	118 (0.3%)	44 (0.2%)	162 (0.2%)	
ANXIETY				<0.001
0	44,026 (97.4%)	28,249 (98.0%)	72,275 (97.6%)	
1	1,173 (2.6%)	574 (2.0%)	1,747 (2.4%)	
MOOD DISORDER				0.035
0	45,192 (100.0%)	28,823 (100.0%)	74,015 (100.0%)	
1	7 (0.0%)	0 (0.0%)	7 (0.0%)	
ADJUSTMENT DISORDER				<0.001
0	45,166 (99.9%)	28,819 (100.0%)	73,985 (100.0%)	
1	33 (0.1%)	4 (0.0%)	37 (0.0%)	
BIPOLAR				<0.001
0	45,097 (99.8%)	28,793 (99.9%)	73,890 (99.8%)	
1	102 (0.2%)	30 (0.1%)	132 (0.2%)	
ADHD				<0.001
0	43,192 (95.6%)	27,817 (96.5%)	71,009 (95.9%)	
1	2,007 (4.4%)	1,006 (3.5%)	3,013 (4.1%)	

VCD, vocal cord dysfunction; ADHD, attention deficit hyperactivity disorder; 0, absence of diagnosis; 1, presence of diagnosis.

### 3.3 VCD vs. Non-VCD Patients

Among patients diagnosed with VCD (*N* = 399), when compared with those without (*N* = 73,623), VCD patients were older (median 14 vs. 9 years) and predominantly female (71% vs. 47%). They were more frequently White/non-Hispanic and demonstrated higher prevalence of comorbid conditions including asthma (41% vs. 16%), allergic rhinitis (20% vs. 11%), and GERD (31% vs. 4%). Psychological diagnoses were also significantly elevated in the VCD group: anxiety (8.5% vs. 2.3%), depression (1.5% vs. 0.2%), adjustment disorder (0.8% vs. 0.0%), and panic attacks (0.8% vs. 0.1%) (Table 3).

**Table 3:** Demographic data, trigger symptoms and psychological diagnosis by VCD diagnosis.

Demographics	Non VCD	VCD	Total	*P* value
		(*N* = 399)	(*N* = 74,022)	
AGE				<0.001
Median (Range)	9.0 (5.0, 21.0)	14.0 (5.0, 20.0)	9.0 (5.0, 21.0)	
Mean (SD)	9.8 (3.9)	13.1 (3.2)	9.8 (3.9)	
SEX				<0.001
F	34,231 (46.5%)	284 (71.2%)	34,515 (46.6%)	
M	39,392 (53.5%)	115 (28.8%)	39,507 (53.4%)	
ETHNICITY				<0.001
N-Miss	3,133	12	3,145	
ANOTHER HISPANIC, LATINO, OR SPANISH ORIGIN	13,464 (19.1%)	32 (8.3%)	13,496 (19.0%)	
NON-HISPANIC OR LATINO	57,026 (80.9%)	355 (91.7%)	57,381 (81.0%)	
RACE				<0.001
WHITE OR CAUCASIAN	42,666 (58.0%)	318 (79.7%)	42,984 (58.1%)	
BLACK OR AFRICAN AMERICAN	13,863 (18.8%)	38 (9.5%)	13,901 (18.8%)	
OTHER	17,094 (23.2%)	43 (10.8%)	17,137 (23.2%)	
ASTHMA				<0.001
0	61,858 (84.0%)	235 (58.9%)	62,093 (83.9%)	
1	11,765 (16.0%)	164 (41.1%)	11,929 (16.1%)	
ALLERGIC RHINITIS				<0.001
0	65,201 (88.6%)	319 (79.9%)	65,520 (88.5%)	
1	8,422 (11.4%)	80 (20.1%)	8,502 (11.5%)	
GERD				<0.001
0	70,537 (95.8%)	275 (68.9%)	70,812 (95.7%)	
1	3,086 (4.2%)	124 (31.1%)	3,210 (4.3%)	
EOSINOPHILIC ESOPHAGISTIS				<0.001
0	73,401 (99.7%)	392 (98.2%)	73,793 (99.7%)	
1	222 (0.3%)	7 (1.8%)	229 (0.3%)	
DEPRESSION				<0.001
0	73,467 (99.8%)	393 (98.5%)	73,860 (99.8%)	
1	156 (0.2%)	6 (1.5%)	162 (0.2%)	
ANXIETY				<0.001
0	71,910 (97.7%)	365 (91.5%)	72,275 (97.6%)	
1	1,713 (2.3%)	34 (8.5%)	1,747 (2.4%)	
ADJUSTMENT DISORDER				<0.001
0	73,589 (100.0%)	396 (99.2%)	73,985 (100.0%)	
1	34 (0.0%)	3 (0.8%)	37 (0.0%)	
PANIC ATTACKS				<0.001
0	73,554 (99.9%)	396 (99.2%)	73,950 (99.9%)	
1	69 (0.1%)	3 (0.8%)	72 (0.1%)	

F, female; M, male; GERD, gastroesophageal reflux disease; 0, absence of diagnosis; 1, presence of diagnosis.

### 3.4 Pre-Pandemic VCD vs. Non-VCD

Pre-pandemic, patients with VCD were older, more frequently female, and more likely to be White/non-Hispanic when compared with non-VCD patients. Psychological diagnoses were significantly more common in the VCD group: anxiety (9.3% vs. 2.6%), depression (1.7% vs. 0.3%), and adjustment disorder (1.0% vs. 0.1%). Attention deficit hyperactivity disorder and panic attacks were rare and not significantly different between groups (Supplementary Table S1).

### 3.5 Pandemic VCD vs. Non-VCD

During the pandemic, findings were consistent with pre-pandemic patterns. Patients with VCD were older, more frequently female, and more often White/non-Hispanic. Psychological diagnoses remained elevated among VCD patients: anxiety (6.2% vs. 2.0%), depression (1.0% vs. 0.1%), and panic attacks (2.1% vs. 0.1%). Both ADHD and adjustment disorder remained uncommon (Supplementary Table S2).

## 4 Discussion

This study demonstrates that the incidence of VCD decreased significantly during the COVID-19 pandemic while maintaining strong and consistent associations with psychological comorbidities despite strong and consistent associations with psychological comorbidities both before and during the pandemic.

The reduction in VCD incidence may reflect decreased exposure to environmental triggers such as school-based stress, athletic participation, and environmental irritants during lockdowns and social restrictions. In parallel, limited access to outpatient services during the pandemic may also have contributed to underdiagnosis of VCD, as children missed opportunities for referral and evaluation.

Psychological diagnoses also declined modestly during the pandemic, which may represent under-recognition due to reduced health care encounters rather than a true decrease. Alternatively, pandemic-related social restrictions may have reduced certain stressors (e.g., academic and performance pressures) while introducing others (e.g., social isolation), resulting in heterogeneous effects on mental health that are difficult to capture through clinical diagnosis alone.

Consistent with prior literature, VCD patients across both time periods were older, more likely female, and more often White/non-Hispanic (7, 8). The persistent association between VCD and psychiatric conditions—including anxiety, depression, and panic attacks—highlights the biopsychosocial nature of the disorder, where psychological distress may influence laryngeal muscle control and symptom perception, while respiratory symptoms can, in turn, exacerbate anxiety and health care utilization. Social and environmental factors, such as pandemic-related disruptions, altered stress exposures, and delays in diagnosis, may further modulate symptom expression and patterns of care. Together, these interconnected influences underscore the need for multidisciplinary diagnostic and management approaches that integrate pulmonary evaluation with mental health assessment and timely access to care.

The higher prevalence of asthma, allergic rhinitis, and GERD in VCD patients supports the notion that VCD often overlaps with or mimics other airway disorders. Such clinical overlap may contribute to diagnostic delay, misclassification as refractory asthma, and prolonged morbidity, increasing health care utilization.

Rather than reiterating the decline in psychological diagnoses, it is important to place these findings within the broader literature. Reviews of youth mental health during COVID-19 have documented increases in depressive and anxiety symptoms, suicidality, and long-term neurocognitive concerns (9). In this context, our observed trends likely reflect challenges in accurately capturing psychiatric morbidity during periods of disrupted health care access, as well as the complex interplay between environmental changes and mental health.

Our finding of decreased incidence of VCD during the pandemic differs from the results reported by Edge et al., who observed an increased incidence of VCD during the same period (12). This discrepancy may reflect differences in health care utilization, referral patterns, or institutional populations or diagnostic thresholds during the pandemic. Unlike the Edge et al. study, we examined the role of psychological comorbidities, which remained strongly associated with VCD across both pre-pandemic and pandemic periods.

In conclusion, the observed reduction in VCD and psychological diagnoses during the COVID-19 pandemic likely reflects a combination of factors rather than a true decline in disease burden. Potential contributors include school closures and reduce academic and athletic stressors, decreased interpersonal contact, limited access to outpatient and specialty care, and delays in diagnostic evaluation. Despite these changes, the persistent association between VCD and psychological comorbidities reinforces the biopsychosocial nature of the disorder. These findings highlight the importance of maintaining access to multidisciplinary care and mental health screening for children with respiratory symptoms, particularly during periods of health system disruption.

## 5 Limitations

Several limitations should be considered. As a retrospective observational study, our findings are subject to potential misclassification and selection bias, particularly given reliance on clinician-documented diagnoses. Residual confounding by unmeasured factors, including socioeconomic stressors and access to care, is possible. Disruptions in health care utilization during the pandemic may have contributed to underdiagnosis of both VCD and psychological conditions (13). In addition, missing data for some variables may limit interpretability, although missingness was limited and addressed through available-case analysis.

## 6 Implications and future directions

Despite these limitations, the findings highlight the importance of maintaining awareness of VCD in pediatric patients presenting with refractory asthma-like symptoms, particularly in those with comorbid psychological conditions. Future prospective studies should examine causal mechanisms linking psychological stress, pandemic-related disruptions, and VCD. Increased access to mental health services may improve recognition and management of VCD in the pediatric population.

## Data Availability

The datasets presented in this article are not readily available because The submitted manuscript utilizes data from pediatric patients enrolled in an IRB-approved study. To protect the long-term privacy of the participants in the study, we will not be able to share the full data for open access. The reasons for this stem from (i) the protection of children as a vulnerable population; (ii) the nature of the IRB approval for the study; and (iii) the relatively small number of participants and the risk of deductive disclosure. Data will be made available on request without charge to individuals completing a simple data use agreement. Requests to access the datasets should be directed to Aledie Navas Nazario, MD, aledie.navasnazario@nemours.org.
