# Supplementary material for: Psychological factors contributing to vocal cord dysfunction in pediatric population pre-pandemic and during pandemic
Source: Front Pediatr. 2026 Feb 18;14:1717883. doi: 10.3389/fped.2026.1717883 (PMC12957235; doi:10.3389/fped.2026.1717883)
Supplement: Supplementary file 1 [file Table1.docx]

Supplemental Table 1. Demographic data and psychological diagnosis by vocal cord dysfunction diagnosis pre-pandemic.

|  | **Non VCD (N=44897)** | **VCD (N=302)** | **Total (N=45199)** | **P value** |
| --- | --- | --- | --- | --- |
| AGE |  |  |  | <0.001 |
| Median (Range) | 9.0 (5.0, 21.0) | 14.0 (5.0, 20.0) | 9.0 (5.0, 21.0) |  |
| Mean (SD) | 9.8 (3.9) | 13.1 (3.2) | 9.8 (3.9) |  |
| SEX |  |  |  | <0.001 |
| F | 20718 (46.1%) | 215 (71.2%) | 20933 (46.3%) |  |
| M | 24179 (53.9%) | 87 (28.8%) | 24266 (53.7%) |  |
| ETHNICITY |  |  |  | <0.001 |
| N-Miss | 1714 | 10 | 1724 |  |
| ANOTHER HISPANIC, LATINO, OR SPANISH ORIGIN | 7954 (18.4%) | 24 (8.2%) | 7978 (18.4%) |  |
| NON-HISPANIC OR LATINO | 35229 (81.6%) | 268 (91.8%) | 35497 (81.6%) |  |
| RACE |  |  |  | <0.001 |
| WHITE OR CAUCASIAN | 26112 (58.2%) | 240 (79.5%) | 26352 (58.3%) |  |
| BLACK OR AFRICAN AMERICAN | 8285 (18.5%) | 25 (8.3%) | 8310 (18.4%) |  |
| OTHER | 10500 (23.4%) | 37 (12.3%) | 10537 (23.3%) |  |
| DEPRESSION |  |  |  | <0.001 |
| 0 | 44784 (99.7%) | 297 (98.3%) | 45081 (99.7%) |  |
| 1 | 113 (0.3%) | 5 (1.7%) | 118 (0.3%) |  |
| ANXIETY |  |  |  | <0.001 |
| 0 | 43752 (97.4%) | 274 (90.7%) | 44026 (97.4%) |  |
| 1 | 1145 (2.6%) | 28 (9.3%) | 1173 (2.6%) |  |
| MOOD DISORDER |  |  |  | 0.83 |
| 0 | 44890 (100.0%) | 302 (100.0%) | 45192 (100.0%) |  |
| 1 | 7 (0.0%) | 0 (0.0%) | 7 (0.0%) |  |
| ADJUSTMENT DISORDER |  |  |  | <0.001 |
| 0 | 44867 (99.9%) | 299 (99.0%) | 45166 (99.9%) |  |
| 1 | 30 (0.1%) | 3 (1.0%) | 33 (0.1%) |  |
| BIPOLAR |  |  |  | 0.11 |
| 0 | 44797 (99.8%) | 300 (99.3%) | 45097 (99.8%) |  |
| 1 | 100 (0.2%) | 2 (0.7%) | 102 (0.2%) |  |
| BEHAVIORAL ISSUES |  |  |  | 0.69 |
| 0 | 44874 (99.9%) | 302 (100.0%) | 45176 (99.9%) |  |
| 1 | 23 (0.1%) | 0 (0.0%) | 23 (0.1%) |  |
| PANIC ATTACKS |  |  |  | 0.22 |
| 0 | 44851 (99.9%) | 301 (99.7%) | 45152 (99.9%) |  |
| 1 | 46 (0.1%) | 1 (0.3%) | 47 (0.1%) |  |
| ADHD |  |  |  | 0.69 |
| 0 | 42902 (95.6%) | 290 (96.0%) | 43192 (95.6%) |  |
| 1 | 1995 (4.4%) | 12 (4.0%) | 2007 (4.4%) |  |

F = female, M = male, ADHD = attention deficit hyperactivity disorder, 0 = absence of diagnosis, 1 = presence of diagnosis
