# Supplementary material for: Psychological factors contributing to vocal cord dysfunction in pediatric population pre-pandemic and during pandemic
Source: Front Pediatr. 2026 Feb 18;14:1717883. doi: 10.3389/fped.2026.1717883 (PMC12957235; doi:10.3389/fped.2026.1717883)
Supplement: Supplementary file 2 [file Table2.docx]

Supplemental Table 2. Demographic data and psychological diagnosis by VCD diagnosis during pandemic.

|  | **Non VCD (N=28726)** | **VCD (N=97)** | **Total (N=28823)** | **P value** |
| --- | --- | --- | --- | --- |
| AGE |  |  |  | <0.001 |
| Median (Range) | 9.0 (5.0, 21.0) | 14.0 (5.0, 18.0) | 9.0 (5.0, 21.0) |  |
| Mean (SD) | 9.8 (4.0) | 13.3 (3.2) | 9.8 (4.0) |  |
| SEX |  |  |  | <0.001 |
| F | 13513 (47.0%) | 69 (71.1%) | 13582 (47.1%) |  |
| M | 15213 (53.0%) | 28 (28.9%) | 15241 (52.9%) |  |
| ETHNICITY |  |  |  | 0.004 |
| N-Miss | 1419 | 2 | 1421 |  |
| ANOTHER HISPANIC, LATINO, OR SPANISH ORIGIN | 5510 (20.2%) | 8 (8.4%) | 5518 (20.1%) |  |
| NON-HISPANIC OR LATINO | 21797 (79.8%) | 87 (91.6%) | 21884 (79.9%) |  |
| RACE |  |  |  | <0.001 |
| WHITE OR CAUCASIAN | 16554 (57.6%) | 78 (80.4%) | 16632 (57.7%) |  |
| BLACK OR AFRICAN AMERICAN | 5578 (19.4%) | 13 (13.4%) | 5591 (19.4%) |  |
| OTHER | 6594 (23.0%) | 6 (6.2%) | 6600 (22.9%) |  |
| DEPRESSION |  |  |  | 0.026 |
| 0 | 28683 (99.9%) | 96 (99.0%) | 28779 (99.8%) |  |
| 1 | 43 (0.1%) | 1 (1.0%) | 44 (0.2%) |  |
| ANXIETY |  |  |  | 0.003 |
| 0 | 28158 (98.0%) | 91 (93.8%) | 28249 (98.0%) |  |
| 1 | 568 (2.0%) | 6 (6.2%) | 574 (2.0%) |  |
| MOOD DISORDER |  |  |  |  |
| 0 | 28726 (100.0%) | 97 (100.0%) | 28823 (100.0%) |  |
| 1 | 0 (0.0%) | 0 (0.0%) | 0 (0.0%) |  |
| ADJUSTMENT DISORDER |  |  |  | 0.91 |
| 0 | 28722 (100.0%) | 97 (100.0%) | 28819 (100.0%) |  |
| 1 | 4 (0.0%) | 0 (0.0%) | 4 (0.0%) |  |
| BIPOLAR |  |  |  | 0.75 |
| 0 | 28696 (99.9%) | 97 (100.0%) | 28793 (99.9%) |  |
| 1 | 30 (0.1%) | 0 (0.0%) | 30 (0.1%) |  |

| BEHAVIORAL ISSUES |  |  |  | 0.82 |
| --- | --- | --- | --- | --- |
| 0 | 28710 (99.9%) | 97 (100.0%) | 28807 (99.9%) |  |
| 1 | 16 (0.1%) | 0 (0.0%) | 16 (0.1%) |  |
| PANIC ATTACKS |  |  |  | <0.001 |
| 0 | 28703 (99.9%) | 95 (97.9%) | 28798 (99.9%) |  |
| 1 | 23 (0.1%) | 2 (2.1%) | 25 (0.1%) |  |
| ADHD |  |  |  | 0.37 |
| 0 | 27725 (96.5%) | 92 (94.8%) | 27817 (96.5%) |  |
| 1 | 1001 (3.5%) | 5 (5.2%) | 1006 (3.5%) |  |

F = female, M = male, ADHD = attention deficit hyperactivity disorder, 0 = absence of diagnosis, 1 = presence of diagnosis
